# Supplementary material for: Expectations and attitudes towards medical artificial intelligence: A qualitative study in the field of stroke
Source: PLoS One. 2023 Jan 11;18(1):e0279088. doi: 10.1371/journal.pone.0279088 (PMC9833517; doi:10.1371/journal.pone.0279088)
Supplement: S1 Appendix — (PDF) [file pone.0279088.s001.pdf]

Appendix 1: Interview guide

1. Participants experiences with stroke and greatest challenges

a. Stroke survivors and family members:
   - How long ago did you have the stroke? How old were you at that time?
   - What are, from your perspective, the biggest challenges after the stroke?

b. Healthcare professionals:
   - How long have you been working in the stroke field?
   - From your perspective, what are the biggest challenges of working with stroke patients?

As mentioned at the beginning, we are particularly interested in your attitudes and expectations towards artificial intelligence. Artificial intelligence can be understood as a collective term for computer applications that can learn independently from data and make predictions. Artificial intelligence can, in this sense, be used to personalize certain services or make personalized recommendations.

2. Understanding of AI and its application

a. **In general:** Do you see applications of artificial intelligence in your daily life?

b. **In healthcare and specifically stroke:** Do you see applications of artificial intelligence in healthcare? Can you think of ways in which artificial intelligence could be used in the field of stroke?

Introduction Vignette: Within Precise4q, we seek to develop AI-based predictive models to make it possible to personalize the prevention, treatment, rehabilitation, and reintegration of stroke patients. The predictions are based on a range of clinical data, such as imaging, as well as medical history and lifestyle information and shall support the clinical decision-making process. The goal is to ensure that each patient receives the treatment that is most promising for him or her. To make accurate predictions, a large amount of data is needed.

To illustrate this, I would like to give you an example: Vignette (See Appendix 2)

3. Expectations regarding AI in stroke

a. Do you consider the presented scenario realistic? Why (not)?
   Prompts:
   i. *Do you have any hopes/concerns in relation to this?*

b. How do you expect that an AI system like this would affect your current situation? (your work as a HCP / your situation as a patient/caregiver)
   Prompts:
   i. *Do you have any hopes/concerns in relation to this?*
   ii. *Where do you see opportunities and/or challenges?*
   iii. *Should patients be informed when artificial intelligence is used?*
   iv. *How might the use of the system impact*
      - *trust between patient and healthcare professional*
      - *responsibility*
      - *fairness*
      - *patient privacy*
      - *autonomy and self-determination*

c. How do you think AI will evolve in the future? Why?
   Prompts:
   i. *Do you have any hopes/concerns in relation to this?*
   ii. *Where do you see opportunities and/or challenges?*

Interview Guide (translated from the German original)
