# Supplementary material for: Expectations and attitudes towards medical artificial intelligence: A qualitative study in the field of stroke
Source: PLoS One. 2023 Jan 11;18(1):e0279088. doi: 10.1371/journal.pone.0279088 (PMC9833517; doi:10.1371/journal.pone.0279088)
Supplement: S2 Appendix — (PDF) [file pone.0279088.s002.pdf]

Appendix 2: Vignette

Remo is 52 years old and works in the accounting department of a large company. His job is stressful, and Remo works a lot of overtime. He has been a smoker for many years and does hardly any sport. Due to his overweight and elevated cholesterol level, his family doctor advised him to pay attention to his diet and do more sports. When Remo is about to leave the office one evening, he suddenly has trouble moving his right arm and leg, and he also finds it difficult to talk. His work colleagues react quickly and call an ambulance. Remo has suffered a stroke.

Arriving at the hospital, the emergency team employs a **new computer program** that uses simulations to predict the success of various treatment options. The program uses Remo’s existing electronic patient file for this purpose. The patient file contains clinical information, such as genetic data, as well as information on Remo's medical history and lifestyle. The rapid response of the emergency team, based on the calculations of the computer program, reduces Remo's risk of suffering permanent function loss.

After his discharge, Remo begins a three-month rehabilitation phase to regain the functions impaired by the stroke through targeted training. In particular, the function of his left leg is still severely impaired, and he has difficulty walking without a walking aid. In addition, Remo has difficulties concentrating and is therefore worried that he will not be able to return to his job. Using **computer-aided simulations**, Remo's treatment team can see how different therapy plans would affect Remo’s recovery progress. Based on these calculations, they design a rehabilitation program tailored to Remo that adapts daily to Remo's condition to achieve an optimal rehabilitation goal.

To ensure that Remo gets the support he needs at home (e.g., shopping or doing the housework), Remo's treatment team again uses the **computer program**. It helps them to assess whether and how intensive support and care Remo needs in order to follow an independent life. The program also calculates Remo's individual risk of suffering another stroke. Based on this information, Remo’s treatment team recommends targeted preventive measures.
